# Agreeing Language in Veterinary Endocrinology (ALIVE): Cushing’s Syndrome and Hypoadrenocorticism—A Modified Delphi-Method-Based System to Create Consensus Definitions

**DOI:** 10.3390/vetsci12080761

**Published:** 2025-08-14

**Authors:** Stijn J. M. Niessen, Ellen N. Behrend, Federico Fracassi, David B. Church, Sue F. Foster, Sara Galac, Carlos Melian, Álan G. Pöppl, Ian K. Ramsey, Nadja S. Sieber-Ruckstuhl, on behalf of the ESVE/SCE membership

**Affiliations:** 1 Royal Veterinary College, University of London, London AL9 7TA, UK; 2 Veterinary Information Network, Davis, CA 95616, USA; 3 Veterinary Specialist Consultations, 1215 JX Hilversum, The Netherlands; 4 Department of Veterinary Clinical Sciences, Faculty of Veterinary Medicine, Michigan State University, East Lansing, MI 48824, USA; 5 College of Veterinary Medicine, Auburn University, Auburn, AL 36849, USA; 6 Department of Veterinary Medical Sciences, University of Bologna, 40064 Bologna, Italy; 7 School of Veterinary Medicine, Murdoch University, Perth 6150, Australia; 8 Department of Clinical Sciences, Faculty of Veterinary medicine, Utrecht University, 3584 CS Utrecht, The Netherlands; 9 Department of Animal Pathology, Veterinary Faculty, University of Las Palmas de Gran Canaria, Arucas, 35413 Las Palmas, Spain; 10 Clínica Veterinaria Atlántico—VetPartners, Pi y Margall, 42, 35006 Las Palmas de Gran Canaria, Spain; 11 Department of Animal Medicine, Faculty of Veterinary, Universidade Federal do Rio Grande do Sul (UFRGS), Porto Alegre 90040-060, RS, Brazil; 12 Brazilian Association of Veterinary Endocrinology (ABEV), São Paulo 01451-001, SP, Brazil; 13 Small Animal Hospital, School of Biodiversity, One Health and Veterinary Medicine, University of Glasgow, 464 Bearsden Road, Bearsden, Glasgow G61 1QH, UK; 14 Clinic for Small Animal Internal Medicine, Vetsuisse Faculty, University of Zurich, CH-8006 Zurich, Switzerland

**Keywords:** consensus, terminology, adrenals, Cushing’s, hypoadrenocorticism, endocrinology

## Abstract

Progress in clinical practice, research, and teaching needs a common language. Agreement among veterinary endocrinologists on definitions of concepts related to Cushing’s syndrome (CS) and hypoadrenocorticism is lacking. After a successful inaugural cycle on diabetes mellitus terminology, project Agreeing Language in Veterinary Endocrinology (ALIVE) held a second cycle, with simplified methodology, and brought together 10 experts of the European Society of Veterinary Endocrinology (ESVE) and the Society of Comparative Endocrinology (SCE). It employed a four-round modified Delphi Method to generate draft definitions and try and achieve consensus. A final round used an endorsement survey of the expert-generated definitions distributed to the ESVE and SCE memberships, seeking a simple majority endorsement. A minimum of 20% membership participation was sought. The 10 experts achieved 100% consensus on the definition of 35 adrenal disease-associated concepts, including disease definitions, diagnostic criteria, and test definitions, a disease classification system for CS and hypoadrenocorticism, and a clinical scoring system for CS. Definitions were subsequently assessed by 78 ESVE and SCE members (26% of combined memberships). All definitions achieved a simple majority, ranging from 83.1 to 100%. ALIVE proved effective in creating a body of terminology for adrenal disease in companion animals, which met the overall approval of a majority of those participating in the endorsement phase. The prospective use of these definitions could help improve comparability and standards for adrenal disease research, education, and clinics.

## Simple Summary

To make progress in the field of hormonal diseases in companion animals, it helps when researchers, clinicians, and educators use the same language. Currently, there is no consensus on basic concepts such as what constitutes the correct definition of diseases affecting the adrenal glands, important hormone-producing glands situated next to the kidneys. This publication reports on the second cycle of a novel project called “Agreeing Language in Veterinary Endocrinology” (ALIVE) that brings experts and those interested in the field together to try and achieve consensus on such disease definitions. The cycle’s methods were adapted from previous ones to improve efficiency and were completed successfully, accomplishing a majority-based consensus. It also delivered agreement on diagnostic criteria for adrenal diseases in companion animals. It is hoped the work will improve education, diagnosis, and treatment in this field, ultimately leading to improvements in the quality of life of animals suffering from adrenal disease.

## 1. Introduction

In research, education, as well as clinical practice, a common language aids the comparability of methodology, results, and conclusions. This, in turn, can facilitate progress in patient outcomes and quality of life. Nonetheless, in many parts of medicine, and indeed veterinary medicine, a specifically agreed common language is lacking, thus preventing progress. Varying disease and diagnostic definitions impede powerful meta-analyses and systematic reviews but also cause confusion in education and clinical practice. To address this issue within veterinary endocrinology, a project called “Agreeing Language in Veterinary Endocrinology (ALIVE)” was founded in 2016 by the European Society of Veterinary Endocrinology (ESVE) and endorsed by the Society of Comparative Endocrinology (SCE); two of the world’s largest veterinary endocrinology societies, bringing together veterinarians with specific interest and knowledge in this area. The first cycle of project ALIVE was successfully completed and previously reported on [1]. The process achieved consensus and resulted in the creation of a body of definitions for use in companion animal diabetology. The inaugural cycle used a modified Delphi approach, variations of which had previously been successful in other areas of medicine and veterinary medicine [2,3,4,5,6,7,8]. The current work describes the results of the second cycle of the same program, which focused on the creation of a body of agreed definitions for adrenal endocrinopathies resulting in Cushing’s syndrome and hypoadrenocorticism in companion animals. The methodology was adjusted by decreasing the number of experts and the number of expert rounds to improve efficiency, increase the number of definitions over which consensus could be achieved, reduce costs, and improve sustainability (e.g., by reducing travelling, reducing email traffic).

## 2. Materials and Methods

**Figure 1:**
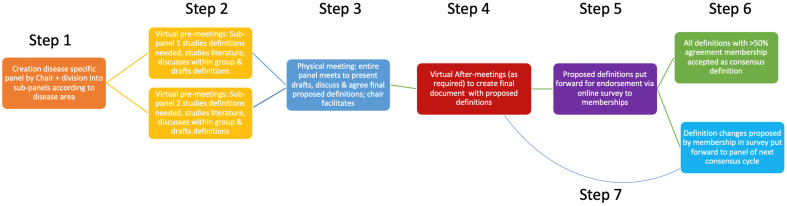
The adapted ALIVE process of Cycle 2 explained in a flow diagram with step numbers showing the order of steps. ALIVE” Agreeing Language in Veterinary Endocrinology.

In this second cycle, the number of experts was reduced from 17 in Cycle 1 to 10 in Cycle 2, and instead of Cycle 1’s two physical expert group meetings, only one was held, resulting in 7 instead of 9 steps in the process (Figure 1) [1]. Overall, the remainder of the steps were unchanged. In summary, group agreement was attempted to be achieved among this group of 10 experts, who volunteered for the cycle after a call-out to the ESVE and SCE membership. The Chair (SN) selected the 10 individuals to ensure appropriate knowledge level, as well as diversity in terms of geographical location and clinical or research settings, in order to ensure wide applicability of the generated definitions (Step 1). As per the Delphi methodology, during each round, the findings of the previous rounds were shared among the group and reflected upon, with the aim of fostering agreement over time. As per Cycle 1, rounds were led by the Chair (SN) as facilitator, and, similar to Cycle 1, anonymity was not part of the process. This lack of anonymity was to increase the probability of reaching agreement relative to the lower likelihood of this being achieved with an anonymised Delphi approach, as has been previously suggested [6].

The panel, aided by the chair, initially divided itself into two sub-panels (with focus on Cushing’s syndrome or hypoadrenocorticism) according to individuals’ professional interest area and each appointed a chair (the sub-chair) (Figure 1; step 1). This sub-division into two smaller groups was thought to aid the efficiency of initial discussions and concept formation, thus enabling the maximum use of the experts’ availability during a limited time.

The sub-panels held a series of virtual meetings (pre-meetings) before the physical meetings to identify, study, and create draft definitions relevant to a specified area within the topic of Cushing’s syndrome and hypoadrenocorticism (Figure 1; step 2). The draft definitions were then presented by the sub-chairs (EB and FF) to the entire group at the start of a series of physical meetings over a two-day period, where all panellists met in person. The draft definitions were then discussed, as well as amended, on the basis of the feedback provided by the rest of the group (Figure 1; step 3). As in Cycle 1, definitions that could not be finalised during the physical meeting, due to time constraints or lingering disagreement, were further discussed and refined in email exchanges and virtual after-meetings (Figure 1; step 4). Arbitrarily, an agreement of at least 75% of panellists was set to be sufficient for the creation of a definition (though 100% agreement was sought).

Finally, panel-agreed definitions were put forward by the chair for endorsement by the entire memberships of both ESVE and SCE through an anonymous online survey sent to all email addresses available through the societies’ secretaries (Figure 1; step 5). Members were contacted on a minimum of three occasions through this route, and participation was encouraged through social media channels as well as during physical society meetings. Possible survey responses offered to members were ‘I endorse this definition’ or ‘I do NOT endorse this definition’ (Figure 1; step 6); all respondents were provided the opportunity to comment on the proposed definitions at the end of the survey. Comments have been stored digitally and will be presented to the panel members of the next cycle. This could lead to future panel members proposing an adaptation to the definitions of this current cycle during a future cycle (Figure 1; step 7). As per Cycle 1, simple majority endorsement (>50% of respondents) qualified the definitions to become official ALIVE-approved terminology for subsequent use in research, education, and clinical practice. The process aimed for a minimum of 20% of memberships to participate in the endorsement phase (survey phase) of the process. Costs associated with the project were covered by funds provided by ESVE as well as external sponsorship. The influence of external sponsors on the content of the process was prohibited. The process of Cycle 2 is summarised in Figure 1.

## 3. Results

Among the 10 panelists, 100% agreement was achieved for 35 adrenal cortex-associated definitions. Definitions were subsequently assessed by 78 ESVE and SCE members (26% of combined memberships). All definitions achieved a simple majority, ranging from 83.1 to 100%. The specific definitions are shown below, including the level of agreement obtained.

### 3.1. Cushing’s Syndrome

#### 3.1.1. Cushing’s Syndrome (Endorsement 72/78)

Cushing’s syndrome is the umbrella term for a range of clinical syndromes that are caused by a chronic excess of glucocorticoid activity, which can be due to a range of endogenous or exogenous steroid hormones.

Comments:

ALIVE discourages the use of the term Cushing’s *disease* or hyperadrenocorticism for the umbrella term of this clinical syndrome.

ALIVE recognises the possibility of pre-clinical or subtle presentations associated with high cortisol concentrations/hypercortisolism.

ALIVE also recognises that demonstrable hypercortisolism is not always associated with or does not always lead to Cushing’s syndrome.

#### 3.1.2. Hypercortisolism (Endorsement 74/78)

Excessive glucocorticoid activity due to cortisol.

#### 3.1.3. Subdiagnostic Cushing’s Syndrome (Endorsement 64/78)

A clinical syndrome in which a dog or cat appears to have Cushing’s syndrome, yet the results of dynamic testing of pituitary-adrenal function fall into appropriate (normal) reference intervals.

Testing requires a normal dexamethasone suppression test (based on blood or urine estimates of corticoid activity) and a normal ACTH stimulation test.

Subdiagnostic Cushing’s syndrome has previously been referred to as Atypical or Occult Cushing’s/Hyperadrenocorticism.

Comments:

ALIVE emphasises that measurement of basal urinary corticoid:creatinine ratio (UCCR) and endogenous ACTH are not dynamic tests of pituitary-adrenal function.

#### 3.1.4. Classification of Cushing’s Syndrome (Endorsement 68/78)

Naturally occurring Cushing’s syndrome
  ACTH-dependent
    Pituitary-dependent hypercortisolism (PDH)
    Ectopic ACTH
    Subdiagnostic Cushing’s syndrome
  ACTH-independent
    Adrenal-dependent hypercortisolism (ADH)
    Aberrant adrenal receptor expression (ectopic and eutopic)
    Subdiagnostic Cushing’s syndrome
Iatrogenic Cushing’s Syndrome

#### 3.1.5. ACTH-Dependent—Pituitary-Dependent Hypercortisolism (PDH) (Endorsement 74/78)

Hypercortisolism occurs due to dysregulated ACTH secretion by the pituitary. This is generally associated with pituitary neoplasia or hyperplasia.

The ALIVE criteria for the diagnosis of PDH are the identification of a set of clinical features attributable to Cushing’s syndrome, including supportive history, physical examination findings, and clinicopathologic test results.

AND demonstration of an excess of cortisol through dynamic testing of pituitary-adrenal function; dynamic testing of pituitary-adrenal function includes a dexamethasone suppression test based on blood or urine or an ACTH stimulation test.

AND ACTH-dependence originating from the pituitary is proven through at least one clear differentiation test result, which includes the following:

A characteristic suppression of an LDDST using blood;
A characteristic suppression of an HDDST using blood;
A characteristic suppression of an HDDST combined with a UCCR measurement;
The absence of suppressed endogenous ACTH concentration;
The absence of an ultrasound examination characteristic of a glucocorticoid-secreting adrenal tumour, using the ALIVE methodology;
Characteristic changes in pituitary morphology on CT or MRI using ALIVE methodology.

Comments:

All diagnostic test methodology needs to comply with ALIVE guidelines (see diagnostic test definitions for more information).

ALIVE emphasises that measurement of basal UCCR and endogenous ACTH is not a dynamic test of pituitary-adrenal function.

The lack of suppression in response to dexamethasone is *not* confirmation of ADH.

Employed cortisol assays should be validated and subjected to quality control; usually, this means tests should be run by reference laboratories and not performed in-house to be reliably accurate.

As there are other diseases that can cause similar clinical signs and false positive test results for Cushing’s syndrome, it is important that, as part of the diagnostic approach, these conditions are excluded (e.g., diabetes mellitus, hypercalcemia, liver insufficiency).

It is accepted that the range of clinical signs of hypercortisolism and what is considered a satisfactory demonstration of cortisol excess varies between individual animals and clinicians.

#### 3.1.6. ACTH-Dependent—Ectopic ACTH (Endorsement 77/78)

A rare form of ACTH-dependent hypercortisolism associated with uncontrolled secretion of ACTH from a non-pituitary site, usually a carcinoid (a malignant tumour of neuroendocrine origin).

The ALIVE criteria for the diagnosis of ACTH-dependent naturally occurring Cushing’s syndrome due to ectopic ACTH secretion (endorsement 66/78):

Clinical signs of Cushing’s syndrome
AND a lack of cortisol suppression in response to dexamethasone
AND markedly increased endogenous ACTH concentration
AND marked bilateral adrenal enlargement
AND pituitary imaging within normal limits
AND the identification of a non-pituitary tumour and resolution of ACTH excess after removal of the tumour.

Comments:

Severe hypokalemia has, thus far, been a typical clinicopathologic finding.

The lungs and pancreas should be evaluated for the presence of the ACTH-secreting tumour; a tumour may not be found, as they can be very small.

#### 3.1.7. ACTH-Dependent—Subdiagnostic Cushing’s Syndrome (Endorsement 75/78)

A clinical syndrome in which the findings of Cushing’s syndrome are due to excess secretion of ACTH, yet an excess of cortisol cannot be proven.

The ALIVE criteria for the diagnosis of ACTH-dependent subdiagnostic Cushing’s syndrome (endorsement 76/78):

Clinical signs of Cushing’s syndrome;
AND other causes of the clinical signs have been eliminated;
AND normal results of all dynamic tests of pituitary-adrenal function;
AND endogenous ACTH concentration is not suppressed;
AND results of abdominal imaging are not characteristic of a glucocorticoid-secreting adrenal tumour using ALIVE criteria.

Comments:

Such cases may be due to the inaccuracy of current reference interval limits.

If these criteria are met, the recommendation is to retest for Cushing’s syndrome in 2–3 months’ time if clinical signs persist.

ALIVE is aware of the use of treatment trials to aid in diagnosis in such situations, especially when pet owners are not coping with the clinical signs or the welfare of the patient is at risk.

ALIVE emphasises that treatment trials can pose significant risks, should not replace the above diagnostic criteria, and, if considered, should only be undertaken when euthanasia is being considered or the patient’s welfare is thought to be at significant risk if treatment is being postponed.

Additionally, any treatment trial should only be conducted in a cautious, well-educated, well-monitored, and supervised manner.

#### 3.1.8. ACTH-Independent—Adrenal-Dependent Hypercortisolism (ADH) (Endorsement 65/78)

Hypercortisolism due to unregulated cortisol secretion by the adrenal cortex. This is generally associated with adrenal neoplasia or hyperplasia.

ALIVE criteria for the diagnosis of ADH (endorsement 77/78):

The identification of a set of clinical features attributable to Cushing’s Syndrome, including supportive history, physical examination findings, and clinicopathologic test results.

AND demonstration of an excess of cortisol through dynamic testing of pituitary-adrenal function; dynamic testing of pituitary-adrenal function can include a dexamethasone suppression test based on blood or urine or an ACTH stimulation test.

AND ACTH-independence confirmed by at least one clear differentiation test result, which includes the following:

Suppressed endogenous ACTH concentration;

An ultrasound examination characteristic of a glucocorticoid-secreting adrenal tumour using ALIVE criteria;

Characteristic changes of adrenal morphology on ultrasound, CT or MRI using ALIVE’s methodology.

Comments:

All diagnostic test methodology needs to comply with ALIVE guidelines (see diagnostic test definitions for more information).

ALIVE emphasises that measurement of basal UCCR and endogenous ACTH is not a dynamic test of pituitary-adrenal function.

A lack of suppression in response to dexamethasone is not confirmation of ADH.

Employed cortisol assays should be validated and subjected to quality control; usually, this means tests should be run by reference laboratories and not performed in-house to be reliably accurate.

As there are other diseases that can cause similar clinical signs and false positive test results for Cushing’s syndrome, it is important that, as part of the diagnostic approach, these conditions are excluded.

The range of clinical signs of hypercortisolism and what is considered a satisfactory demonstration of cortisol excess varies between individual animals and clinicians.

#### 3.1.9. ACTH-Independent—Aberrant Adrenal Receptor Expression (Ectopic and Eutopic) (Endorsement 78/78)

A form of ACTH-independent hypercortisolism caused by excess cortisol secretion due to aberrant expression of receptors in the adrenal cortex. A subtype of aberrant adrenal expression is food-dependent hypercortisolism, due to receptors for gastrointestinal hormones. The presence of receptors for gastric inhibitory polypeptide (GIP) is the only aberrant expression currently demonstrated in dogs. In this form of Cushing’s syndrome, food ingestion induces GIP release from the small intestine, which binds to its receptor in the adrenal gland and causes excess cortisol secretion.

ALIVE criteria for the diagnosis of the food-dependent form of aberrant adrenal receptor expression (endorsement 77/78):

Clinical signs of Cushing’s syndrome.
AND normal results of dynamic testing of pituitary-adrenal function.
AND suppressed endogenous ACTH concentration.
AND increased UCCR after ingestion of a meal.
AND no evidence of one adrenal gland being smaller than normal.
AND pituitary imaging within normal limits.
AND a supportive octreotide test, where octreotide administration prevents food-induced cortisol elevation.

#### 3.1.10. ACTH-Independent—Subdiagnostic Cushing’s Syndrome (Endorsement 75/78)

A clinical syndrome in which the findings of Cushing’s syndrome are due to secretion by an adrenal tumour of a non-cortisol hormone that has glucocorticoid activity, e.g., corticosterone, progesterone.

ALIVE criteria for the diagnosis of ACTH-independent subdiagnostic Cushing’s syndrome (endorsement 77/78):

Clinical signs of Cushing’s syndrome

AND normal or below normal results of dynamic testing of pituitary-adrenal function

And ONE of the following:

Results of abdominal imaging characteristic of a glucocorticoid-secreting adrenal tumour, using ALIVE criteria, and suppressed endogenous ACTH concentration.

Comments

If these criteria are fulfilled, identification of the hormone(s) being secreted by the adrenal tumour is not necessary for effective clinical management.

#### 3.1.11. Iatrogenic Cushing’s Syndrome (Endorsement 68/78)

A form of Cushing’s syndrome caused by chronic administration of systemic or topical glucocorticoids.

ALIVE criteria for the diagnosis of iatrogenic Cushing’s syndrome (endorsement 64/78)

A history of receiving, or having recently received, chronic exogenous glucocorticoid therapy

AND lack of cortisol secretion as demonstrated by an ACTH stimulation test AND resolution of clinical signs with withdrawal of the exogenous glucocorticoids.

Comments:

A similar syndrome can be caused by the administration of exogenous progestins.

Sudden cessation of exogenous glucocorticoids should be avoided, given the risk of hypoadrenocorticism.

#### 3.1.12. ALIVE Goals of Treatment of Naturally Occurring Cushing’s Syndrome (Endorsement 71/78)

To optimise quality of life, to eliminate clinical signs (which can be quantified using the ALIVE Cushing’s Clinical Score, Table 1), and to reduce long-term complications and mortality.

These are achieved by eliminating the source of either ACTH or autonomous adrenal hormone excess, or at least, controlling excess adrenal hormone secretion.

Treatment ideally should be considered only if there are clinical signs consistent with naturally occurring Cushing’s syndrome and when the disease is confirmed by endocrine testing.

Comments:

Differentiating between forms of naturally occurring Cushing’s syndrome is highly desirable in order to optimise management strategies and prognosticate.

Iatrogenic glucocorticoid excess is treated by gradual cessation of exogenous glucocorticoid administration.

#### 3.1.13. ALIVE Cushing’s Clinical Score (Endorsement 72/78)

**Table 1:** ALIVE Cushing’s Clinical Score.

Factor	Score ^1^
Drinking—compared to before the onset of Cushing’s 0 = Normal (drinks the same amount or less) 1 = Mild (some increase in drinking) 2 = Moderate (notable increase in drinking) 3 = Severe (constantly seen to be drinking)	…
Urination—compared to before the onset of Cushing’s 0 = Normal (urinates the same amount or less) 1 = Mild (some increase noted by owner) 2 = Moderate (notable increase noted by owner) 3 = Severe (constantly needs to be let out to urinate)	…
Appetite—compared to before the onset of Cushing’s 0 = Normal or decreased appetite (if decreased appetite, exclude hypoadrenocorticism, macroadenoma, or concurrent disease) 1 = Mild polyphagia (finishes eagerly) 2 = Moderate polyphagia (finishes eagerly and begs for more) 3 = Severe polyphagia (obsessed with food)	…
Appearance 0 = Normal 1 = Mild abnormalities (slightly poor hair and skin quality) 2 = Moderate abnormalities (poor hair and skin quality with hair loss and/or some muscle loss/pot belly) 3 = Severe abnormalities (substantial hair loss and/or noticeable muscle loss/pot belly)	…
Attitude/activity—compared to before the onset of Cushing’s 0 = Normal 1 = Mild decrease (not quite themselves) 2 = Moderate decrease (quieter and less active and/or panting more than normal) 3 = Severe decrease (very quiet, dull, and weak and/or noticeable increase in panting) (consider hypoadrenocorticism and macroadenoma in the ill dog with Cushing’s)	…
TOTAL SCORE out of 15 ^2^	…

^1^ range total score: 0–15. ^2^ Treatment aim: lowest score possible without unacceptably high risk of hypoadrenocorticism.

### 3.2. Hypoadrenocorticism

#### 3.2.1. Hypoadrenocorticism (Endorsement 73/78)

Hypoadrenocorticism is an umbrella term for naturally occurring or iatrogenic disorders that cause reduced adrenal cortex function, resulting in glucocorticoid deficiency, mineralocorticoid deficiency, or both.

Comments:

The term Addison’s (disease) is synonymous with “primary hypoadrenocorticism” and does not include other forms of hypoadrenocorticism.

#### 3.2.2. Primary Hypoadrenocorticism (Endorsement 76/78)

Primary hypoadrenocorticism is a condition caused by adrenocortical injury and either naturally occurring (most commonly immune-mediated) or iatrogenic due to surgery (e.g., bilateral adrenalectomy) or drugs (e.g., mitotane, trilostane).

Comments:

There are acute and chronic presentations, and, in chronic presentations, animals sometimes have periods without overt clinical signs. Addison’s (disease) is a synonym for “primary hypoadrenocorticism”; ALIVE encourages the use of Primary Hypoadrenocorticism since it is more descriptive and, thus, less open to confusion.

Less common causes of naturally occurring adrenal injury include neoplasia, infarction, infection, haemorrhage, and necrosis.

#### 3.2.3. Secondary Hypoadrenocorticism (Endorsement 66/78)

Glucocorticoid or, less likely, mineralocorticoid deficiency due to lack of ACTH or renin, respectively. Secondary glucocorticoid-deficient hypoadrenocorticism can be naturally occurring or iatrogenic due to surgery (e.g., hypophysectomy, post-adrenalectomy of a cortisol-producing adrenal tumour) or abrupt discontinuation of drugs with glucocorticoid activity, including progestins.

#### 3.2.4. Hyponatremic and/or Hyperkalemic Hypoadrenocorticism (Endorsement 76/78)

This is defined as hypoadrenocorticism with hyperkalemia and/or hyponatremia.

This is due to primary hypoadrenocorticism.

Comments:

This form has previously been referred to as typical hypoadrenocorticism.

ALIVE recognises a current inability to accurately measure aldosterone concentrations. Therefore, clinical status is currently classified by sodium and potassium abnormalities and not by measurement of aldosterone concentration.

Depending on the magnitude of hyponatremia and/or hyperkalemia, measurement of endogenous ACTH to differentiate primary and secondary hypoadrenocorticism may be considered.

Isolated aldosterone deficiency has rarely been described. These cases have reference interval cortisol concentrations. Diagnosis constitutes documenting low blood sodium concentration and high blood potassium, and exclusion of all other causes. For these cases, aldosterone measurement would be ideal; however, ALIVE recognises a current inability to accurately measure aldosterone concentrations.

Dogs with confirmed isolated mineralocorticoid deficiency may also progress to become glucocorticoid-deficient.

#### 3.2.5. Eunatremic, Eukalemic Hypoadrenocorticism (Endorsement 78/78)

This is defined as hypoadrenocorticism with normal serum concentrations of potassium and sodium. This could be due to primary or secondary hypoadrenocorticism. In order to prove that primary hypoadrenocorticism is present, endogenous ACTH concentration must be measured and be normal or above the reference interval.

Comments:

This form has previously been referred to as atypical hypoadrenocorticism.

ALIVE recognises a current inability to accurately measure aldosterone concentrations. Therefore, clinical status is currently classified by sodium and potassium abnormalities and not by measurement of aldosterone concentration.

#### 3.2.6. ALIVE Criteria for the Diagnosis of Glucocorticoid Deficiency (Endorsement 76/78)

Cortisol concentrations pre- and post-ACTH are within or less than the lower quartile of the reference interval for basal cortisol; e.g., if the basal cortisol reference interval is 30–120 nmol/L (1.1–4.4 ug/dL), a post-ACTH cortisol concentration of 53 nmol/L (1.9 ug/dL) or less provides a diagnosis of hypoadrenocorticism.

Crucially, confirmation needs to occur that no exogenous glucocorticoids or progestogens are being or have been administered by any route, including topical, prior to diagnosis.

Comments:

ALIVE emphasises that topical routes of glucocorticoid therapy include ophthalmic and otic, among others.

ALIVE recommends that employed cortisol assays should be validated and subjected to quality control; usually, this means tests should be run by reference laboratories and not performed in-house to be reliably accurate.

#### 3.2.7. ALIVE Criteria for the Diagnosis of Primary Hypoadrenocorticism (Endorsement 73/78)

Cortisol concentrations pre- and post-ACTH are within or less than the lower quartile of the reference interval for basal cortisol; e.g., if the basal cortisol reference interval is 30–120 nmol/L (1.1–4.4 ug/dL), a post-ACTH cortisol concentration of 53 nmol/L (1.9 ug/dL) or less provides a diagnosis of hypoadrenocorticism.

Documentation of a normal or above normal endogenous ACTH. Measurement of endogenous ACTH should ideally be performed. ALIVE finds it reasonable to abstain from measurement of endogenous ACTH given the more common nature of primary versus secondary hypoadrenocorticism. However, secondary hypoadrenocorticism cannot be diagnosed without an endogenous ACTH measurement.

Crucially, confirmation needs to occur that no exogenous glucocorticoids or progestogens are being or have been administered by any route, including topical, prior to diagnosis.

Isolated aldosterone deficiency has rarely been described. These cases have reference interval cortisol concentrations. Diagnosis constitutes documenting low blood sodium concentration and high blood potassium, and exclusion of all other causes. For these cases, aldosterone measurement would be ideal; however, ALIVE recognises a current inability to accurately assess aldosterone concentrations.

Dogs with confirmed isolated mineralocorticoid deficiency may also progress to become glucocorticoid-deficient.

Comments:

ALIVE emphasises that topical routes of glucocorticoid administration include ophthalmic and otic, among others.

ALIVE recommends that employed cortisol assays should be validated and subjected to quality control; usually, this means tests should be run by reference laboratories and not performed in-house to be reliably accurate.

#### 3.2.8. ALIVE Criteria for the Diagnosis of Secondary Hypoadrenocorticism (Endorsement 68/78)

Cortisol concentrations pre- and post-ACTH are within or less than the lower quartile of the reference interval for basal cortisol; e.g., if the basal cortisol reference interval is 30–120 nmol/L (1.1–4.4 ug/dL), a post-ACTH cortisol concentration of 53 nmol/L (1.9 ug/dL) or less provides a diagnosis of hypoadrenocorticism.

Endogenous ACTH concentration is below the reference interval.

Comments:

If cortisol deficiency is known to be due to surgery (removal of pituitary or adrenal tumour or recent cessation of a drug with glucocorticoid activity), measurement of endogenous ACTH to confirm the diagnosis of secondary hypoadrenocorticism is not necessary.

ALIVE recommends that employed cortisol assays should be validated and subjected to quality control; usually, this means tests should be run by reference laboratories and not performed in-house to be reliably accurate.

#### 3.2.9. Critical Illness-Related Corticosteroid Insufficiency (CIRCI) (Endorsement 73/78)

A syndrome referred to as CIRCI (previously called relative adrenal insufficiency or RAI) is reported in the literature. Definitive evidence for the existence of CIRCI is lacking. Some critically ill patients, especially those who are volume-depleted, hypotensive, and vasopressor-resistant, may benefit from the administration of drugs with glucocorticoid activity. However, if CIRCI exists, the criteria for diagnosis and guidelines for treatment of the syndrome are currently unknown.

#### 3.2.10. Adrenal Crisis, Formerly Known as Addisonian Crisis (Endorsement 73/78)

This is a severe, acute presentation of hypoadrenocorticism, which usually includes weakness, hypovolemia, and hypotension in addition to anorexia and vomiting; abdominal pain may be present. If untreated, an adrenal crisis can progress to shock and death.

Comments:

With Addison’s (disease) being a synonym for “primary hypoadrenocorticism” and an adrenal crisis also occurring with other forms of hypoadrenocorticism (i.e., secondary hypoadrenocorticism), ALIVE encourages the use of “Adrenal crisis” instead of “Addisonian crisis”.

#### 3.2.11. ALIVE Goals of Acute Treatment of an Adrenal Crisis (Endorsement 76/78)

These goals are the correction of

Clinical signs;

Hypotension;

Hypovolaemia;

Electrolyte imbalances, most importantly hyperkalaemia;

Hypoglycaemia (if present);

Anaemia (if severe and life-threatening).

Comment:

ALIVE emphasises the need to avoid rapid correction of hyponatremia in order to minimise the risk of osmotic myelinolysis.

#### 3.2.12. ALIVE Goals of Chronic Treatment of Hypoadrencorticism (Endorsement 73/78)

These goals are

Lack of clinical signs;

Normal or near normal electrolyte concentrations;

Specifically avoiding excessive, chronic glucocorticoid supplementation (resulting in iatrogenic Cushing’s syndrome, weight gain, and/or any biomarkers of excess glucocorticoid activity such as increased blood alkaline phosphatase activity (ALP), marked lymphopenia, etc.) and mineralocorticoid supplementation (resulting in hypertension, hypokalemia, and/or hypernatremia).

### 3.3. Definitions for Adrenal Assessment Tests

#### 3.3.1. Resting Cortisol (Endorsement 68/78)

Measurement of a single serum or plasma cortisol concentration by obtaining a blood sample at any time of day.

Comments:

Resting cortisol is not a screening test for Cushing’s syndrome. Resting cortisol can be used as a screening test to rule out hypoadrenocorticism; a low value cannot prove the presence of hypoadrenocorticism and should be followed by an ACTH stimulation test for confirmation of the disease.

For all tests, cut-off values have to be established by each laboratory.

Measurement of cortisol using in-house assays is discouraged, and the use of reference laboratories is encouraged. In the clinical setting, in which tubes may be underfilled, the use of EDTA-plasma significantly increases the measured concentration of cortisol.

#### 3.3.2. Urinary Corticoid:Creatinine Ratio (UCCR) (Endorsement 73/78)

Measurement of urinary corticoid (cortisol and its metabolites) excretion. The ratio is calculated by dividing urinary corticoid concentration by urinary creatinine concentration.

Comments:

The sample used should be the first morning urine sample.

The UCCR is, at best, a poor screening test for Cushing’s syndrome, as its sensitivity can be below 70% depending on the assay employed.

ALIVE recommends that the urine be collected at home >2 days after a visit to a veterinary clinic or stressful event.

The UCCR should never be considered as an effective test for the diagnosis of Cushing’s syndrome, as its specificity is less than 70%

For all tests, cut-off values have to be established by each laboratory.

#### 3.3.3. Urinary Corticoid: Creatinine Ratio (UCCR) Dexamethasone Suppression Test (Endorsement 73/78)

Defined as the determination of UCCR on two consecutive days, followed by administration of oral dexamethasone (0.1 mg/kg q8h) on day 2 and a collection of a third sample for a UCCR on the third day.

Comments:

The UCCR dexamethasone suppression test is a screening and differentiating test.

The samples used should be the first morning urine samples.

ALIVE recommends the urine be collected at home >2 days after a visit to a veterinary clinic or stressful event.

For all tests, cut-off values have to be established by each laboratory.

#### 3.3.4. Low-Dose Dexamethasone Suppression Test (LDDST) (Endorsement 67/78)

Dexamethasone (0.01 mg/kg in dogs and 0.1 mg/kg in cats) is injected intravenously, and serum/plasma cortisol is measured before injection and 4 and 8 h thereafter.

Comments:

The LDDST can be a screening and differentiating test.

Despite the existence of alternative dosage and timing recommendations, ALIVE encourages the future use of the above protocol for standardisation and comparability purposes.

For all tests, cut-off values have to be established by each laboratory.

Measurement of cortisol by in-house assays is discouraged, and the use of reference laboratories is encouraged.

In the clinical setting in which tubes may be underfilled, use of EDTA-plasma significantly increases the measured concentration of cortisol.

#### 3.3.5. High-Dose Dexamethasone Suppression Test (HDDST) (Endorsement 78/78)

Dexamethasone (0.1 mg/kg in dogs and 1.0 mg/kg in cats) is injected intravenously, and serum/plasma cortisol is measured before injection and 4 and 8 h thereafter.

Comments:

The HDDST can be a differentiating test, although it cannot prove the existence of ADH.

Despite the existence of alternative dosage and timing recommendations, ALIVE encourages the future use of the above protocol for standardisation and comparability purposes.

For all tests, cut-off values have to be established by each laboratory.

Measurement of cortisol by in-house assays is discouraged, and the use of reference laboratories is encouraged.

In the clinical setting in which tubes may be underfilled, use of EDTA-plasma significantly increases the measured concentration of cortisol.

#### 3.3.6. ACTH Stimulation Test (Endorsement 76/78)

The ACTH stimulation test is performed by administering synthetic ACTH (preferred dose: 5 μg/kg in dogs and 125 μg/cat), and serum/plasma cortisol concentrations are measured before and 60 min (dogs) or 60 and 90 min (cats) after ACTH administration.

Comments:

The ACTH stimulation test is a screening test for hypoadrenocorticism and naturally occurring Cushing’s syndrome, and the only test that can document iatrogenic Cushing’s syndrome.

Intravenous use of the synthetic ACTH is considered standard; though intramuscular use has also been shown to be effective, for standardisation and comparability purposes, ALIVE recommends intravenous administration, where possible. Several synthetic ACTH preparations exist, with tetracosactide and cosyntropin being the most frequently used; ALIVE recommends the use of preparations with sufficient evidence base for efficacy.

Several dosages of synthetic ACTH are being used; ALIVE recommends the use of a minimum of 5 μg/kg (dogs) intravenously.

Several timings for post-ACTH sampling have been reported (including several additional samples for cats); ALIVE recommends the above 60 min in dogs and, at least, 60 min and 90 min in cats, for standardisation and comparability purposes.

Measurement of cortisol by in-house assays is discouraged, and the use of reference laboratories is encouraged.

In the clinical setting in which tubes may be underfilled, the use of EDTA-plasma significantly increases the measured concentration of cortisol.

#### 3.3.7. Endogenous ACTH Concentration (eACTH) (Endorsement 78/78)

A single EDTA-plasma sample is collected for measurement of endogenous ACTH.

Comments:

Measurement of eACTH is a differentiating test and cannot be used as a screening test.

As eACTH is a fragile hormone, sampling and transport recommendations must be closely followed.

For all tests, cut-off values have to be established by each laboratory.

Measurement of eACTH by a reference laboratory enrolled in an external quality assurance program is encouraged.

#### 3.3.8. Adrenal Imaging (in the Context of Differentiating Tests) (Endorsement 77/78)

Imaging of the adrenal glands can be used as a differentiating test in the context of Cushing’s syndrome. The dorsoventral measurement of the caudal pole thickness of either adrenal gland in a sagittal plane is the best dimension for evaluating size.

Comments:

A single reference interval for a normal measurement is not appropriate; body size must be taken into consideration.

This is not a screening test; therefore, the finding of normal adrenal glands does not exclude Cushing’s syndrome.

Finding enlarged adrenal glands without a clinical suspicion of Cushing’s syndrome and positive endocrine testing does not constitute a diagnosis of Cushing’s syndrome.

ACTH-dependent forms usually present with bilateral symmetrical adrenomegaly, but adrenal asymmetry may also occur.

Adrenal asymmetry, contralateral atrophy, or destruction of the normal tissue architecture are suggestive of ACTH-independent Cushing’s.

Vascular invasion and possible liver metastasis suggest malignancy.

#### 3.3.9. Pituitary Imaging (in the Context of Differentiating Tests for Cushing’s Syndrome) (Endorsement 68/78)

CT or MRI can be used to image the pituitary. The use of contrast enhancement or a dynamic study increases the sensitivity of the techniques.

Comments:

The finding of an apparently normal pituitary on CT and/or MRI does not exclude the possibility of a pituitary abnormality, tumour or hyperplasia.

This is not a screening test; therefore, the finding of an enlarged pituitary without a clinical suspicion of Cushing’s syndrome and positive endocrine testing does not constitute a diagnosis of Cushing’s syndrome either.

#### 3.3.10. Hair and Salivary Cortisol (Endorsement 65/78)

Not enough evidence-based data are currently available to recommend routine use of these tests to diagnose Cushing’s syndrome.

## 4. Discussion

The amended ALIVE protocol, with fewer experts and fewer discussion sessions, still managed to deliver a series of consensus definitions using the previously established level of agreement (>50% of responding ESVE and SCE membership agreeing, with >20% of overall membership responding). In addition, markedly more definitions (37) were generated and agreed upon than previously (16). This suggests that the amended protocol is preferable for achieving its mission. The disadvantage of generating more definitions during one single cycle is posed by possible decreased participation during the final survey phase of the memberships, due to so-called survey fatigue or the process simply taking too much time to study the proposed definitions. Indeed, during cycle one, 34.4–36.6% of the membership participated (a range is given since answers for some definitions were not provided by some responding members—the survey settings have since been adjusted, making providing an answer mandatory for cycle 2), as opposed to 26% during the current cycle. Nonetheless, the threshold of >20% of membership participation was still achieved.

During the review process of this paper, it was highlighted that improvements could be made to the agreed terminology. It was highlighted that for a diagnosis of ACTH-dependent naturally occurring Cushing’s syndrome due to ectopic ACTH secretion, tumour identification is currently required according to the ALIVE criteria. Nonetheless, the same criteria also acknowledge that a tumour may not be found. It was suggested that this tension could undermine the practical utility of the criteria. This could be improved upon by introducing a hierarchy of evidence or allowing for a presumptive diagnosis in cases with high clinical suspicion. The reviewer’s feedback is an example of how it is hoped that the presented definitions do not remain static and can be improved over time. Feedback is therefore actively welcomed using a dedicated email address alive@esve.org (Step 7 of the ALIVE process). Amendments can then be considered and approved by future cycles if deemed appropriate or necessary.

Clinicians, researchers, and educators are encouraged to start using the ALIVE definitions of this latest cycle. Only through actual use of the ALIVE definitions during clinical, scientific, and teaching activities will this process truly achieve its mission. In this regard, the experience from the first ALIVE cycle yields reason for optimism. Since its publication, 40 original peer-reviewed articles have used and cited one or more definitions of this cycle on diabetes mellitus definitions (source: Scopus 11 June 2025), and its definitions were also included in the latest editions of frequently consulted veterinary textbooks (e.g., Ettinger’s Textbook of Veterinary Internal Medicine) [9,10,11,12,13,14,15,16,17]. In the largest online veterinary community, the Veterinary Information Network (www.vin.com), project ALIVE was mentioned in 131 discussions, which related to gaining advice on a patient with diabetes mellitus (source: www.vin.com, accessed on 5 June 2025). Furthermore, the ALIVE definitions were actively mentioned during presentations and in the proceedings of several large veterinary conferences (e.g., ECVIM congress 2023, 2024; ACVIM congress 2023, 2024; BVA Live 2024). It is hoped that the definitions agreed through the current cycle on adrenal disease concepts will have a similarly positive and widespread influence on the field.

## Data Availability

No new data, other than those presented in this manuscript, were generated.

## Abbreviations

The following abbreviations are used in this manuscript:

ALIVE	Agreeing Language in Veterinary Endocrinology
ESVE	European Society of Veterinary Endocrinology
SCE	Society of Comparative Endocrinology
